# Pair density wave at high magnetic fields in cuprates with charge and spin orders

**DOI:** 10.1038/s41467-020-17138-z

**Published:** 2020-07-03

**Authors:** Zhenzhong Shi, P. G. Baity, J. Terzic, T. Sasagawa, Dragana Popović

**Affiliations:** 1 National High Magnetic Field Laboratory, Florida State University, Tallahassee, FL 32310 USA; 2 Department of Physics, Florida State University, Tallahassee, FL 32306 USA; 3 Materials and Structures Laboratory, Tokyo Institute of Technology, Kanagawa, 226-8503 Japan; 4 Present Address: Department of Physics, Duke University, Durham, NC 27708 USA; 5 Present Address: James Watt School of Engineering, University of Glasgow, Glasgow, Scotland G12 8QQ UK

**Keywords:** Quantum fluids and solids, Superconducting properties and materials

## Abstract

In underdoped cuprates, the interplay of the pseudogap, superconductivity, and charge and spin ordering can give rise to exotic quantum states, including the pair density wave (PDW), in which the superconducting (SC) order parameter is oscillatory in space. However, the evidence for a PDW state remains inconclusive and its broader relevance to cuprate physics is an open question. To test the interlayer frustration, the crucial component of the PDW picture, we perform transport measurements on charge- and spin-stripe-ordered La_1.7_Eu_0.2_Sr_0.1_CuO_4_ and La_1.48_Nd_0.4_Sr_0.12_CuO_4_ in perpendicular magnetic fields (*H*_⊥_), and also with an additional field applied parallel to CuO_2_ layers (*H*_∥_). We detect several phenomena predicted to arise from the existence of a PDW, including an enhancement of interlayer SC phase coherence with increasing *H*_∥_. These data also provide much-needed transport signatures of the PDW in the regime where superconductivity is destroyed by quantum phase fluctuations.

## Abstract (web summary)

Among the exotic phases in underdoped cuprates, the evidence of a pair density wave (PDW) remains inconclusive. Here, Shi et al. report transport signatures consistent with the presence of PDW pairing correlations that compete with uniform superconductivity in two underdoped cuprate superconductors.

## Introduction

The origin of the cuprate pseudogap regime has been a long-standing mystery. The richness of experimental observations^1^ and the instability of underdoped cuprates towards a variety of ordering phenomena, such as periodic modulations of charge density discovered in all families of hole-doped cuprates^2^, have raised the possibility that putative pair density wave (PDW) correlations^3,4^ may be responsible for the pseudogap regime^5,6^. In order to distinguish between different scenarios, the most intriguing open question is what happens at low $T\ll {T}_{{\rm{c}}}^{0}$ (here ${T}_{{\rm{c}}}^{0}$ is the *H* = 0 superconducting (SC) transition temperature) and high *H*_⊥_, when SC order is destroyed by quantum phase fluctuations^6^ and short-range charge orders are enhanced^7–9^. However, the experimental evidence for a PDW state remains scant and largely indirect in the first place.

A PDW SC state was proposed^4,10^ to explain the suppression of the interlayer (*c*-axis) Josephson coupling (or dynamical layer decoupling) apparent in the *H* = 0 anisotropic transport^11^ in La_1.875_Ba_0.125_CuO_4_, as well as in optical measurements in La_1.85−*y*_Nd_*y*_Sr_0.15_CuO_4_ when the Nd concentration was tuned into the stripe-ordered regime^12^. The dynamical layer decoupling was observed also in the presence of an applied *H*_⊥_, in La_1.905_Ba_0.095_CuO_4_ (ref. ^13^) and La_2−*x*_Sr_*x*_CuO_4_  (ref. ^14^). In La_2−*x*−*y*_(Ba,Sr)_*x*_(Nd,Eu)_*y*_CuO_4_ compounds near *x* = 1/8, charge order appears in the form of stripes, which are separated by regions of oppositely phased antiferromagnetism (spin stripes)^5^ at *T* < *T*_SO_ < *T*_CO_; here *T*_SO_ and *T*_CO_ are the onsets of spin and charge stripes, respectively. In La_2−*x*_Sr_*x*_CuO_4_  at *x* = 0.10, spin stripe order is induced^15^ by applying *H*_⊥_. The dynamical layer decoupling was thus attributed^4,10^ to a PDW SC state^3,10^, such that the spatially modulated SC order parameter, with zero mean, occurs most strongly within the charge stripes, but the phases between adjacent stripes are reversed (antiphase). Since stripes are rotated by 90° from one layer to next, antiphase superconductivity within a plane strongly frustrates the interlayer SC phase coherence^5^, leading to an increase in anisotropy. This effect is reduced by doping away from *x* = 1/8, but *H*_⊥_ can lead to dynamical layer decoupling as static stripe order is stabilized by a magnetic field.

To obtain more definitive evidence of the existence of a PDW, recent experiments have focused on testing various theoretical predictions^5^. For example, transport measurements on La_1.875_Ba_0.125_CuO_4_ have employed *H*_⊥_ high enough to decouple the planes and then to suppress the SC order within the planes, with the results consistent with pair correlations surviving in charge stripes^16^; Josephson junction measurements^17^ on La_1.875_Ba_0.125_CuO_4_ devices support the prediction of a charge-4*e* SC condensate, consistent with the presence of a PDW state; an additional charge order was detected^18^ in Bi_2_Sr_2_CaCu_2_O_8_ by scanning tunneling microscopy (STM) at very low ${H}_{\perp }/{T}_{{\rm{c}}}^{0}\,\lesssim \, 0.1$ T/K, consistent with a PDW order that emerges within the halo region surrounding a vortex core once a uniform SC order is sufficiently suppressed by *H*_⊥_. However, alternative explanations are still possible, and additional experiments are thus needed to search for a PDW and explore its interplay with other orders in the pseudogap regime^6^.

Therefore, we measure transport in La_2−*x*−*y*_Sr_*x*_(Nd,Eu)_*y*_CuO_4_ compounds, which have the same low-temperature structure as La_2−*x*_Ba_*x*_CuO_4_, over an unprecedented range of *T* down to $T/{T}_{{\rm{c}}}^{0}\,\lesssim\, 0.003$ and fields up to $H/{T}_{{\rm{c}}}^{0} \sim 10$ T/K. We combine linear in-plane resistivity *ρ*_ab_, nonlinear in-plane transport or voltage–current (*V*–*I*) characteristics, and the anisotropy ratio *ρ*_c_/*ρ*_ab_ (here *ρ*_c_ is the out-of-plane resistivity) to probe both charge and vortex matter on single crystals with the nominal composition La_1.7_Eu_0.2_Sr_0.1_CuO_4_  and La_1.48_Nd_0.4_Sr_0.12_CuO_4_  (see “Methods” section); the former is away from *x* = 1/8 and thus the stripe order is weaker^5^. We find signatures of dynamical layer decoupling in both *H* = 0 and with increasing *H*_⊥_, consistent with the presence of a PDW. However, a key proposed test of this interpretation involves relieving the interlayer frustration through the application of an in-plane magnetic field^5,10^. In particular, since *H*_∥_ can reorient the spin stripes in every other plane^19–21^, a consequence of a PDW would be an enhancement of interplane coherence, or a reduced anisotropy. This is precisely what we test and observe.

## Results

### Anisotropy in *H* = 0

**Fig. 1:**
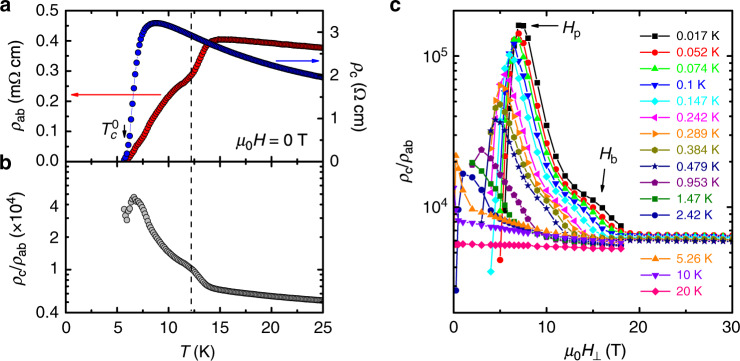
Evolution of the anisotropy in La_1.7_Eu_0.2_Sr_0.1_CuO_4_ with ***T*** and ***H***_⊥_. **a** *ρ*_ab_(*T*) and *ρ*_c_(*T*), and **b** the anisotropy ratio *ρ*_c_/*ρ*_ab_(*T*), in zero field. The vertical dashed line indicates where SC correlations are established in the planes, resulting in the enhancement of the anisotropy; *ρ*_c_ continues to grow with decreasing *T*. **c** *ρ*_c_/*ρ*_ab_ vs. *H*_⊥_ at different *T*, as shown. Arrows show the positions of the anisotropy peak *H*_p_, or the decoupling field, as well as *H*_b_, where the anisotropy is enhanced. The method to determine *H*_b_ more precisely is described in Supplementary Fig. 1.

In both La_1.7_Eu_0.2_Sr_0.1_CuO_4_  and La_1.48_Nd_0.4_Sr_0.12_CuO_4_, *ρ*_c_ and *ρ*_ab_ vanish at the same ${T}_{{\rm{c}}}^{0}$ within the error (see "Methods” section; see also Supplementary Note 1), indicating the onset of 3D superconductivity, similar to La_2−*x*_Sr_*x*_CuO_4_  (e.g. ref. ^22^). The initial drop of *ρ*_ab_(*T*) with decreasing *T* (Fig. 1a) is accompanied by an enhancement of the anisotropy (Fig. 1b), which continues to increase by almost an order of magnitude as *T* is lowered further towards ${T}_{{\rm{c}}}^{0}$. These data look remarkably similar to those on La_1.875_Ba_0.125_CuO_4_ (ref. ^11^) that motivated theoretical proposals for a PDW SC state in striped cuprates: the initial, high-*T* enhancement of the anisotropy is understood to reflect the establishment of SC correlations in CuO_2_ planes.

### Evolution of the anisotropy and *ρ*_ab_ with *H*_⊥_ and *T*

**Fig. 2:**
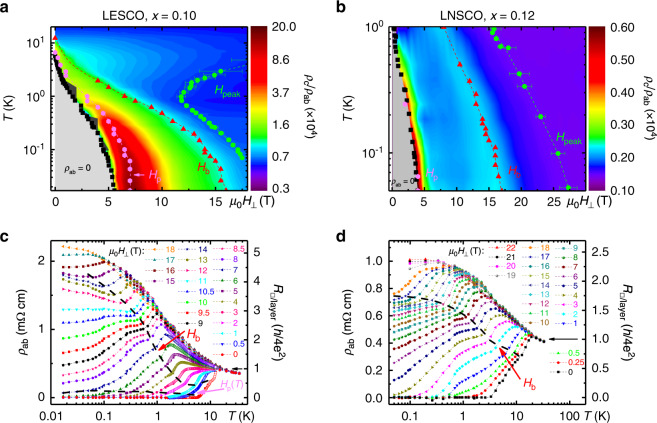
Anisotropy and the in-plane resistivity for different ***T*** and ***H***_⊥_. The color map in **a** and **b** shows *ρ*_c_/*ρ*_ab_ in La_1.8−*x*_Eu_0.2_Sr_*x*_CuO_4_ (LESCO) with *x* = 0.10 (data from Fig. 1c) and La_1.6−*x*_Nd_0.4_Sr_*x*_CuO_4_ (LNSCO) with *x* = 0.12, respectively. Black squares: *T*_c_(*H*_⊥_); *ρ*_ab_ = 0 for all *T* < *T*_c_(*H*_⊥_). Green dots: *H*_peak_(*T*), i.e. fields above which the in-plane MR changes from positive to negative; it has been established^23^ that *H*_peak_(*T*) ~ *H*_c2_(*T*), i.e. the upper critical field. The error bars reflect the uncertainty in defining the MR peak within our experimental resolution (see inset of Supplementary Fig. 2a for an example; also see Supplementary Fig. 6a and ref. ^23^ for the raw MR data). Pink dots: *H*_p_(*T*), the layer decoupling field; red triangles: *H*_b_(*T*), where SC correlations are established in the planes as the SC transition is approached from the normal state. *ρ*_ab_(*T*) of **c** La_1.7_Eu_0.2_Sr_0.1_CuO_4_  and **d** La_1.48_Nd_0.4_Sr_0.12_CuO_4_  for several *H*_⊥_, as shown. Open symbols in **c** show the data from another run. Short-dashed lines guide the eye. The *H*_b_(*T*) values obtained from the anisotropy are represented by the black dashed lines, as shown. The lower black dashed line in **c** corresponds to the layer decoupling field, *H*_p_(*T*). In **d**, *H*_p_(*T*) ≳ *H*_c_(*T*) [or *T*_c_(*H*_⊥_)]. Black arrows in **c** and **d** show that the splitting of the *ρ*_ab_(*T*) curves for different *H*_⊥_ becomes pronounced when *R*_□/layer_ ≈ *R*_Q_ = *h*/(2*e*)^2^.

The evolution of *ρ*_c_/*ρ*_ab_(*T*) with *H*_⊥_ is shown in Fig. 1c. The anisotropy at the highest *T* = 20 K is *ρ*_c_/*ρ*_ab_ ~ 6000 and practically independent of *H*_⊥_. However, as *T* is lowered below ${T}_{{\rm{c}}}^{0}$, *ρ*_c_/*ρ*_ab_ develops a distinctly nonmonotonic behavior as a function of *H*_⊥_. At *T* = 0.017 K, for example, the anisotropy increases with *H*_⊥_ by over an order of magnitude before reaching a peak (*ρ*_c_/*ρ*_ab_ > 10^5^) at *H*_⊥_ = *H*_p_, signifying decoupling of or the loss of phase coherence between the planes. However, strong SC correlations persist in the planes for *H*_⊥_ > *H*_p_: here *ρ*_c_/*ρ*_ab_ decreases with *H*_⊥_ to *H*_⊥_-independent values, comparable to those at high *T*, for the highest *H*_⊥_ > 20 T. This is in agreement with previous evidence^23^ that the *H*_⊥_ > 20 T region corresponds to the normal state. A smooth, rapid decrease of the anisotropy for *H*_⊥_ > *H*_p_ is interrupted by a bump or an enhancement in *ρ*_c_/*ρ*_ab_, centered at *H*_b_. Therefore, the behavior of *ρ*_c_/*ρ*_ab_ is qualitatively the same whether the SC transition is approached from either (1) the high-*T* normal state by lowering *T* in *H* = 0 (Fig. 1b) or (2) the high-*H*_⊥_ normal state by reducing *H*_⊥_ at a fixed *T* (Fig. 1c). These results thus suggest that the enhancement of the anisotropy near *H*_b_(*T*) may be attributed to the establishment of SC correlations in the planes as the SC transition is approached from the high-field normal state.

**Fig. 3:**
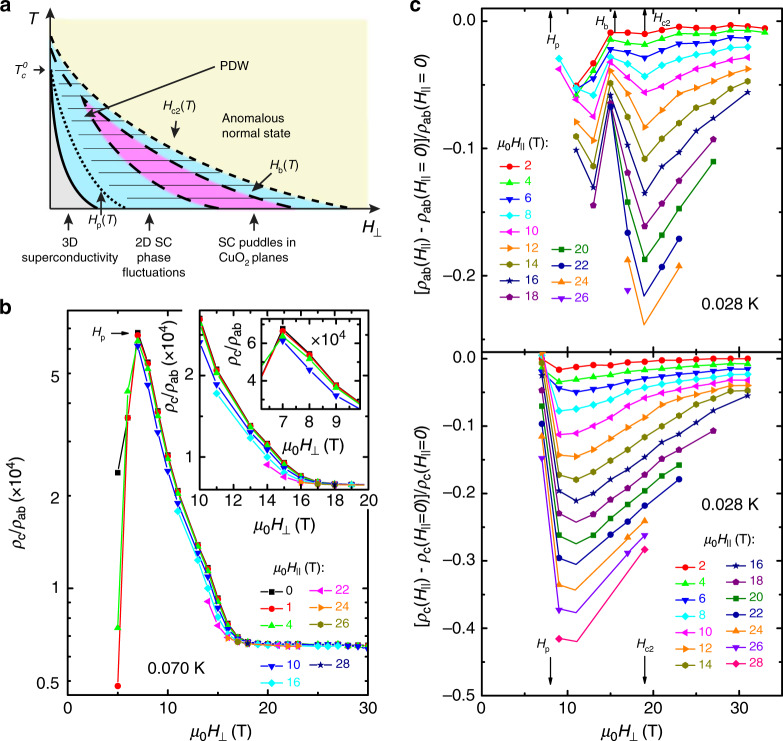
**Evidence for a PDW from anisotropic transport**. **a** Schematic *T*–*H*_⊥_ phase diagram. *H*_⊥_ suppresses the 3D superconductivity (gray) and decouples (dotted line) the CuO_2_ layers at *H*_⊥_ = *H*_p_(*T*). Strong SC phase fluctuations persist in the planes up to *H*_c2_(*T*) (short-dashed line). The behavior in the pink region, the precursors of which appear already in *H* = 0 at $T\,> \,{T}_{{\rm{c}}}^{0}$ (see dashed lines), is consistent with the presence of SC puddles in CuO_2_ planes. An additional, in-plane field enhances the interlayer coupling for *H*_p_(*T*) < *H*_⊥_ < *H*_c2_(*T*), consistent with the presence of PDW correlations (thin hatched lines). Except for the thick solid line, other lines do not represent phase boundaries, but correspond to finite-temperature crossovers. **b** *ρ*_c_/*ρ*_ab_ (for La_1.7_Eu_0.2_Sr_0.1_CuO_4_  in-plane sample B1) vs. *H*_⊥_ for different *H*_∥_, as shown, at *T* = 0.070 K. Larger inset: Enlarged view of the same data shows the suppression of the anisotropy by *H*_∥_ for *H*_p_ < *H*_⊥_ < *H*_c2_. Smaller inset: *ρ*_c_/*ρ*_ab_ is reduced by  ~10% near *H*_p_ by *H*_∥_ up to 10 T. **c** The corresponding [*ρ*_ab_(*H*_∥_)/*ρ*_ab_(*H*_∥_ = 0) − 1] (top, sample B1) and [*ρ*_c_(*H*_∥_)/*ρ*_c_(*H*_∥_ = 0) − 1] (bottom) vs. *H*_⊥_ at *T* = 0.028 K for different *H*_∥_, as shown. In all panels, solid lines guide the eye.

This picture is supported by the comparison of *ρ*_c_/*ρ*_ab_, as a function of *T* and *H*_⊥_, with the behavior of *ρ*_ab_(*T*) for a fixed *H*_⊥_, as shown in Fig. 2 for both La_1.7_Eu_0.2_Sr_0.1_CuO_4_ and La_1.48_Nd_0.4_Sr_0.12_CuO_4_. The *ρ*_ab_(*T*) data were extracted from the in-plane magnetoresistance (MR) measurements (ref. ^23^, Supplementary Fig. 2a; unless stated otherwise, the results are shown for La_1.7_Eu_0.2_Sr_0.1_CuO_4_  sample B, see “Methods” section); the raw *ρ*_c_(*H*) data are shown in Supplementary Fig. 2b, c. In Fig. 2a, b, we also include *T*_c_(*H*_⊥_), as well as *H*_peak_, the position of the peak in the in-plane MR (see e.g. Supplementary Fig. 2a), which corresponds^23^ to the upper critical field *H*_c2_ in these materials (see also Supplementary Note 1). Indeed, at a fixed *T*, *ρ*_c_/*ρ*_ab_ starts to increase as *H*_⊥_ is reduced below *H*_peak_. This is followed by an enhancement of *ρ*_c_/*ρ*_ab_ near *H*_⊥_ = *H*_b_, corresponding to the initial, metallic-like drop of *ρ*_ab_(*T*) as the SC transition is approached from the normal state for a fixed *H*_⊥_ (Fig. 2c, d). The behavior of both materials is similar, except that the layer decoupling field *H*_p_(*T*) ≳ *H*_c_(*T*) [or *T*_c_(*H*_⊥_)] in La_1.48_Nd_0.4_Sr_0.12_CuO_4_, as expected^5^ for a stronger stripe order and frustration of interlayer coupling for *x* ≈ 1/8. Therefore, practically all the data in Fig. 2c, d, i.e. for *H*_⊥_ > *H*_p_, involve “purely” 2D physics, with no communication between the planes. The striking splitting of the *ρ*_ab_(*T*) curves in both materials (ref. ^23^, Fig. 2c, d), into either metallic-like (i.e. SC-like) or insulating-like, when the normal state sheet resistance *R*_□/layer_ ≈ *R*_Q_, where *R*_Q_ = *h*/(2*e*)^2^ is the quantum resistance for Cooper pairs, further supports this conclusion: it agrees with the expectations for a 2D superconductor–insulator transition (SIT) driven by quantum fluctuations of the SC phase^24^. In addition, as previously noted^23^, the two-step *ρ*_ab_(*T*) is reminiscent of that in granular films of conventional superconductors and systems with nanoscale phase separation, including engineered Josephson junction arrays, where they are generally attributed to the onset of local (e.g. in islands or puddles) and global, 2D superconductivity. Similarities to the behavior of various SC 2D systems^25,26^ thus suggest the formation of SC islands as *H*_⊥_ is reduced below *H*_b_ at a fixed *T* (e.g. Fig. 2a, b), i.e. at the initial, metallic-like drop of *ρ*_ab_(*T*) for a fixed *H*_⊥_ (*H*_b_ dashed line in Fig. 2c, d). Additional evidence in support of this interpretation, such as the *V*–*I* that is characteristic of a viscous vortex liquid in the puddle regime, is discussed in Supplementary Note 2 (also, Supplementary Figs. 3–5). Therefore, at low *T*, the increasing *H*_⊥_ destroys the superconductivity in the planes by quantum phase fluctuations of Josephson-coupled SC puddles. The evolution of this puddle region with *T* can be traced to the initial, metallic-like drop of *ρ*_ab_(*T*) at $T\,> \,{T}_{{\rm{c}}}^{0}$ in *H* = 0 (see *H*_b_ dashed line in Fig. 2c, d, and Supplementary Figs. 3 and 4). Further increase of *H*_⊥_ at low *T* then leads to the loss of SC phase coherence in individual puddles and, eventually, transition to the high-field normal state. These results are summarized in the sketch of the phase diagram, shown in Fig. 3a.

Our experiments are thus consistent with the presence of local PDW correlations (in puddles) at $T\,> \,{T}_{{\rm{c}}}^{0}$ in *H* = 0, which are overtaken by the uniform d-wave superconductivity at low $T\,<\,{T}_{{\rm{c}}}^{0}$. In transport, the PDW SC order becomes apparent when the uniform d-wave order is sufficiently weakened by *H*_⊥_: it appears beyond the melting field of the vortex solid, within the vortex liquid regime, i.e. in the regime of strong 2D phase fluctuations. Higher fields *H*_p_ are needed to decouple the layers in La_1.7_Eu_0.2_Sr_0.1_CuO_4_  than in La_1.48_Nd_0.4_Sr_0.12_CuO_4_, since it is farther away from *x* = 1/8. In the *T* → 0 limit and for even higher *H*_⊥_ (<*H*_c2_), the system seems to break up into SC puddles with the PDW order. However, the final and key test of the presence of a PDW requires the application of a suitable perturbation, in particular *H*_∥_, to reduce the interlayer frustration and decrease the anisotropy^5^.

### Effects of *H*_∥_ on the anisotropy

We have performed angle-dependent measurements of both *ρ*_ab_(**H**) and *ρ*_c_(**H**), where the angle *θ* is between **H** and the crystalline *c*-axis. This has allowed us to explore the effect of in-plane fields ${H}_{\parallel }=H\sin \theta$ at different ${H}_{\perp }=H\cos \theta$, i.e. fields parallel to the *c*-axis, discussed above. The angle-dependent *ρ*_ab_(**H**) was measured also on another La_1.7_Eu_0.2_Sr_0.1_CuO_4_  sample (sample B1, see “Methods” section; Supplementary Fig. 8); the results are qualitatively the same on both samples. Figure 3b illustrates the effect of *H*_∥_ on *ρ*_c_/*ρ*_ab_ at low *T* = 0.070 K on sample B1 (see Supplementary Fig. 9a–d for the raw *ρ*_c_ and *ρ*_ab_ data at different *T*). Clearly, there is no effect of *H*_∥_ for *H*_⊥_ > *H*_c2_ (*T* = 0.070 K) ≈ 17.5 T. Since *H*_∥_ should break up Cooper pairs through the Zeeman effect, this confirms the absence of any observable remnants of superconductivity above the previously identified^23^ *H*_c2_ (along *c*-axis). In contrast, for *H*_p_ ≤ *H*_⊥_ < *H*_c2_, *H*_∥_ reduces the anisotropy, which is precisely what is expected in the presence of a PDW SC state if the dominant effect of *H*_∥_ is to reorient the spin stripes^10^.

To understand exactly how *H*_∥_ affects the anisotropy, we also investigate Δ*ρ*_ab_ = *ρ*_ab_(*H*_∥_) − *ρ*_ab_(*H*_∥_ = 0) and Δ*ρ*_c_ = *ρ*_c_(*H*_∥_) − *ρ*_c_(*H*_∥_ = 0) at different *H*_⊥_ (Fig. 3c and Supplementary Fig. 8d for sample B1; Supplementary Fig. 9e–h for sample B). It is obvious that *ρ*_ab_ is reduced by *H*_∥_ for all *H*_⊥_, which is the opposite of what would be expected if pair-breaking was dominant. The suppression of *ρ*_ab_ is weaker for those *H*_⊥_ where the superconductivity is stronger, e.g. near *H*_b_ ~ 15 T in Fig. 3c, and conversely, it is most pronounced above *H*_c2_, indicating that the dominant effect of *H*_∥_ is not related to superconductivity. In fact, it occurs most strongly in the two regimes where *ρ*_ab_(*H*_⊥_) exhibits hysteretic behavior at low *T* (Supplementary Figs. 3 and 6); the latter is attributed to the presence of domains with spin stripes (see also Supplementary Note 2 and Supplementary Fig. 7). This observation, therefore, further supports the conclusion that the main effect of *H*_∥_ is the reorientation of spin stripes in every other plane^19–21^ (see also Supplementary Note 3). The suppression of *ρ*_ab_ by *H*_∥_ seems to vanish at experimentally inaccessible *H*_⊥_, where the anomalous, insulating-like $\mathrm{ln}\,(1/T)$ dependence observed in the field-induced normal state also appears to vanish^23^, suggesting that the origin of the $\mathrm{ln}\,(1/T)$ behavior might be related to the presence of short-range spin stripes. As the spin stripes in every other plane are rotated by *H*_∥_, in the PDW picture the interlayer frustration should be suppressed, leading to a decrease in *ρ*_c_. This is precisely what is observed (Fig. 3c). The anisotropy ratio *ρ*_c_/*ρ*_ab_ is reduced (Fig. 3b) because the effect of *H*_∥_ on *ρ*_c_ is relatively stronger than on *ρ*_ab_. Similar results are obtained in La_1.48_Nd_0.4_Sr_0.12_CuO_4_  (Supplementary Fig. 10): here the reduction in *ρ*_c_ is weaker than in La_1.7_Eu_0.2_Sr_0.1_CuO_4_  and *ρ*_ab_ is not affected within the experimental resolution, both consistent with the stronger pinning of stripe order at *x* = 1/8 (see also Supplementary Note 3). Nevertheless, the reduction of *ρ*_c_/*ρ*_ab_ by *H*_∥_ is comparable to that in La_1.7_Eu_0.2_Sr_0.1_CuO_4_  (Fig. 3b). Therefore, by applying an in-plane magnetic field, as proposed theoretically^5,10^, our measurements confirm the presence of a PDW in both La_1.7_Eu_0.2_Sr_0.1_CuO_4_  and La_1.48_Nd_0.4_Sr_0.12_CuO_4_. The effects of *H*_∥_ are observable up to $T\,> \,{T}_{{\rm{c}}}^{0}$ (i.e. *T* ~ *T*_SO_ in La_1.7_Eu_0.2_Sr_0.1_CuO_4_: Supplementary Fig. 9), providing additional evidence for the PDW correlations in *H* = 0 at $T\,> \,{T}_{{\rm{c}}}^{0}$, as sketched in Fig. 3a.

## Discussion

Our findings are thus consistent with the presence of local, PDW pairing correlations that compete with the uniform SC order at ${T}_{{\rm{c}}}^{0}\,<\,T\,<\,(2-6){T}_{{\rm{c}}}^{0}$, and become dominant at intermediate *H*_⊥_ as *T* → 0. Our results also provide an explanation for the surprising, and a priori counterintuitive, observation^23^ that *H*_c2_ in La_1.48_Nd_0.4_Sr_0.12_CuO_4_  (*H*_c2_ ~ 25 T) is higher than that in La_1.7_Eu_0.2_Sr_0.1_CuO_4_  (*H*_c2_ ~ 20 T), even though its zero-field ${T}_{{\rm{c}}}^{0}$ is lower because of stronger stripe correlations. It is clear, though, that it is precisely because of the stronger stripe order and the presence of a more robust PDW SC state at *x* ≈ 1/8 that the superconductivity persists to higher fields as *T* → 0.

In summary, by probing the previously inaccessible high ${H}_{\perp }/{T}_{{\rm{c}}}^{0}$ and *T* → 0 regime dominated by quantum phase fluctuations and by testing a theoretical prediction, we have obtained evidence consistent with the existence of a PDW state in the La-214 family of cuprates with stripes. Our observation of several signatures of a PDW in the regime with many vortices (i.e. a vortex liquid) is also consistent with the STM evidence^18^ for a PDW order that emerges in vortex halos. Since the observed PDW correlations extend only up to *T* ≪ *T*_pseudogap_ and not beyond *H*_c2_(*T*), our results do not support a scenario in which the PDW correlations are responsible for the pseudogap.

## Methods

### **Samples**

Several single crystal samples of La_1.8−*x*_Eu_0.2_Sr_*x*_CuO_4_ with a nominal *x* = 0.10 and La_1.6−*x*_Nd_0.4_Sr_*x*_CuO_4_ with a nominal *x* = 0.12 were grown by the traveling-solvent floating-zone technique^27^. The high homogeneity of the crystals was confirmed by several techniques, as discussed in detail elsewhere^23^. It was established that the samples were at least as homogeneous as those previously reported in the literature and, in fact, the disorder in our La_1.7_Eu_0.2_Sr_0.1_CuO_4_  crystals was significantly lower than that in other studies. We note that the trivial possibility that the two-step SC transition observed at *H* = 0 (e.g. Fig. 2c, d for La_1.7_Eu_0.2_Sr_0.1_CuO_4_  and La_1.48_Nd_0.4_Sr_0.12_CuO_4_, respectively) may be due to an extrinsic inhomogeneity, e.g. the presence of two regions with different values of ${T}_{{\rm{c}}}^{0}$, is clearly ruled out also by the behavior of d*ρ*_ab_/d*T* with *H*_⊥_ (Supplementary Figs. 3a, 4, and 8b). In particular, both materials exhibit a reentrant metallic-like behavior at high *H*_⊥_, below *H*_c2_ (e.g. see the reentrant darker blue color band for La_1.48_Nd_0.4_Sr_0.12_CuO_4_). This is the opposite of what is expected in case of two different ${T}_{{\rm{c}}}^{0}$ values corresponding to different doping levels, where one would expect a gradual suppression of superconductivity with *H*_⊥_, i.e. no reentrance.

The samples were shaped as rectangular bars suitable for direct measurements of the in-plane and out-of-plane resistance. In La_1.7_Eu_0.2_Sr_0.1_CuO_4_, detailed measurements of *ρ*_ab_ were performed on sample B with dimensions 3.06 × 0.53 × 0.37 mm^3^ (*a* × *b* × *c*); *ρ*_c_ was measured on a bar with 0.34 × 0.41 × 1.67 mm^3^. The in-plane La_1.48_Nd_0.4_Sr_0.12_CuO_4_  crystal with dimensions 3.82 × 1.19 × 0.49 mm^3^ was cut along the crystallographic [110] and [1$\bar{1}$0] axes, i.e. at a 45° angle with respect to *a* and *b*. A bar with 0.21 × 0.49 × 3.9 mm^3^ (*a* × *b* × *c*) was used to measure *ρ*_c_ in La_1.48_Nd_0.4_Sr_0.12_CuO_4_. The behavior of these samples remained stable for the duration of numerous experimental runs carried out in different cryostats and magnets (see below) that were needed for this study. After  ~3 years, the low-*T* properties of sample B changed, resulting in a quantitatively different *T*–*H*_⊥_ phase diagram (Supplementary Fig. 8b); this is why we consider it a different sample (B1). The phase diagram of sample B1 seems to be intermediate to those of sample B (Supplementary Fig. 3a) and La_1.48_Nd_0.4_Sr_0.12_CuO_4_  (Supplementary Fig. 4). Electrical contacts were made by evaporating Au on polished crystal surfaces such that, for current contacts, the two opposing faces were fully covered with Au to ensure a uniform current flow, while multiple voltage contacts made on the side faces were narrow enough to minimize the error in the absolute values of the resistance. This was followed by annealing in air at 700 °C. The data are shown for the voltage contacts separated by 1.53 mm for La_1.7_Eu_0.2_Sr_0.1_CuO_4_  and 2.00 mm for La_1.48_Nd_0.4_Sr_0.12_CuO_4_  in-plane samples; 0.47 mm for La_1.7_Eu_0.2_Sr_0.1_CuO_4_  and 1.26 mm for La_1.48_Nd_0.4_Sr_0.12_CuO_4_  out-of-plane samples. Dupont 6838 Ag paste was used to attach gold leads (≈25 μm thick) to the samples, with a subsequent heat treatment at 450 °C in the flow of oxygen for 15 min. The room *T* contact resistances were <0.1 Ω for La_1.7_Eu_0.2_Sr_0.1_CuO_4_, i.e. <0.5 Ω for La_1.48_Nd_0.4_Sr_0.12_CuO_4_. The properties of the samples, including the values of ${T}_{{\rm{c}}}^{0}$, did not depend on the choice of voltage contacts used in the measurements, as expected in the absence of extrinsic (i.e. compositional) inhomogeneity.

${T}_{{\rm{c}}}^{0}$ was defined as the temperature at which the linear resistivity becomes zero, i.e. falls below the experimental noise floor (~0.5 mΩ). For the in-plane samples, ${T}_{{\rm{c}}}^{0}=(5.7\pm 0.3)$ K for La_1.7_Eu_0.2_Sr_0.1_CuO_4_  and ${T}_{{\rm{c}}}^{0}=(3.6\pm 0.4)$ K for La_1.48_Nd_0.4_Sr_0.12_CuO_4_; the out-of-plane resistivity *ρ*_c_ vanishes at (5.5 ± 0.3) K for La_1.7_Eu_0.2_Sr_0.1_CuO_4_  and (3.4 ± 0.5) K for La_1.48_Nd_0.4_Sr_0.12_CuO_4_. In La_1.7_Eu_0.2_Sr_0.1_CuO_4_, *T*_SO_ ~ 15 K, *T*_CO_ ~ 40 K (ref. ^28^), and the pseudogap temperature *T*_pseudogap_ ~ 175 K (ref. ^29^); in La_1.48_Nd_0.4_Sr_0.12_CuO_4_, *T*_SO_ ~ 50 K, *T*_CO_ ~ 70 K (ref. ^30^), and *T*_pseudogap_ ~ 150 K (ref. ^29^).

### Measurements

The standard four-probe ac method (~13 Hz) was used for measurements of the sample resistance, with the excitation current (density) of 10 μA (~5 × 10^−3^ and  ~2 × 10^−3^ A cm^−2^ for La_1.7_Eu_0.2_Sr_0.1_CuO_4_ and La_1.48_Nd_0.4_Sr_0.12_CuO_4_, respectively) for the in-plane samples and 10 nA (~7 × 10^−6^ and  ≲10^−5^ A cm^−2^ for La_1.7_Eu_0.2_Sr_0.1_CuO_4_  and La_1.48_Nd_0.4_Sr_0.12_CuO_4_, respectively) for the out-of-plane samples. d*V*/d*I* measurements were performed by applying a dc current bias (density) down to 2 μA (~1 × 10^−3^ and  ~4 × 10^−4^ A cm^−2^ for La_1.7_Eu_0.2_Sr_0.1_CuO_4_  and La_1.48_Nd_0.4_Sr_0.12_CuO_4_  in-plane samples, respectively) and a small ac current excitation *I*_ac_ ≈ 1 μA (~13 Hz) through the sample and measuring the ac voltage across the sample. For each value of *I*_dc_, the ac voltage was monitored for 300 s and the average value recorded. The relaxations of d*V*/d*I* with time, similar to that in Supplementary Fig. 7, were observed only at the lowest *T* ~ 0.016 K. Even then, the change of d*V*/d*I* during the relaxation, reflected in the error bars for the *T* = 0.017 K data in Supplementary Fig. 3c, was much smaller than the change of d*V*/d*I* with *I*_dc_. The data that were affected by Joule heating at large dc bias were not considered. To reduce the noise and heating by radiation in all measurements, a 1 kΩ resistor in series with a *π* filter [5 dB (60 dB) noise reduction at 10 MHz (1 GHz)] was placed in each wire at the room temperature end of the cryostat.

The experiments were conducted in several different magnets at the National High Magnetic Field Laboratory: a dilution refrigerator (0.016 K ≤ *T* ≤ 0.7 K) and a ^3^He system (0.3 K ≤ *T* ≤ 35 K) in superconducting magnets (*H* up to 18 T), using 0.1–0.2 T min^−1^ sweep rates; a portable dilution refrigerator (0.02 K ≤ *T* ≤ 0.7 K) in a 35 T resistive magnet, using 1 T min^−1^ sweep rate; and a ^3^He system (0.3 K ≤ *T* ≤ 20 K) in a 31 T resistive magnet, using 1–2 T min^−1^ sweep rates. Below  ~0.06 K, it was not possible to achieve sufficient cooling of the electronic degrees of freedom to the bath temperature, a common difficulty with electrical measurements in the mK range. This results in a slight weakening of the *ρ*_ab_(*T*) curves below  ~0.06 K for all fields. We note that this does not make any qualitative difference to the phase diagram (Supplementary Fig. 3a). The fields were swept at constant temperatures, and the sweep rates were low enough to avoid eddy current heating of the samples. The MR measurements with **H**∥**c** were performed also by reversing the direction of **H** to eliminate by summation any Hall effect contribution to the resistivity. Moreover, since Hall effect had not been explored in these materials in large parts of the phase diagrams studied here, we have also carried out detailed measurements of the Hall effect; the results of that study will be presented elsewhere^31^.

The resistance per square per CuO_2_ layer *R*_□/layer_ = *ρ*_ab_/*l*, where *l* = 6.6 Å  is the thickness of each layer.

## Supplementary information

Supplementary Information

Peer Review File

## Data Availability

The data that support the findings of this study are available within the paper and the Supplementary Information. Additional data related to this paper may be requested from the authors.
