# Supplementary material for: Pair density wave at high magnetic fields in cuprates with charge and spin orders
Source: Nat Commun. 2020 Jul 3;11:3323. doi: 10.1038/s41467-020-17138-z (PMC7335199; doi:10.1038/s41467-020-17138-z)
Supplement: Supplementary file 1 — Supplementary Information [file 41467_2020_17138_MOESM1_ESM.pdf]

Supplementary Information for

Pair density wave at high magnetic fields in cuprates with charge and spin orders

Shi, *et al.*

Supplementary Figures

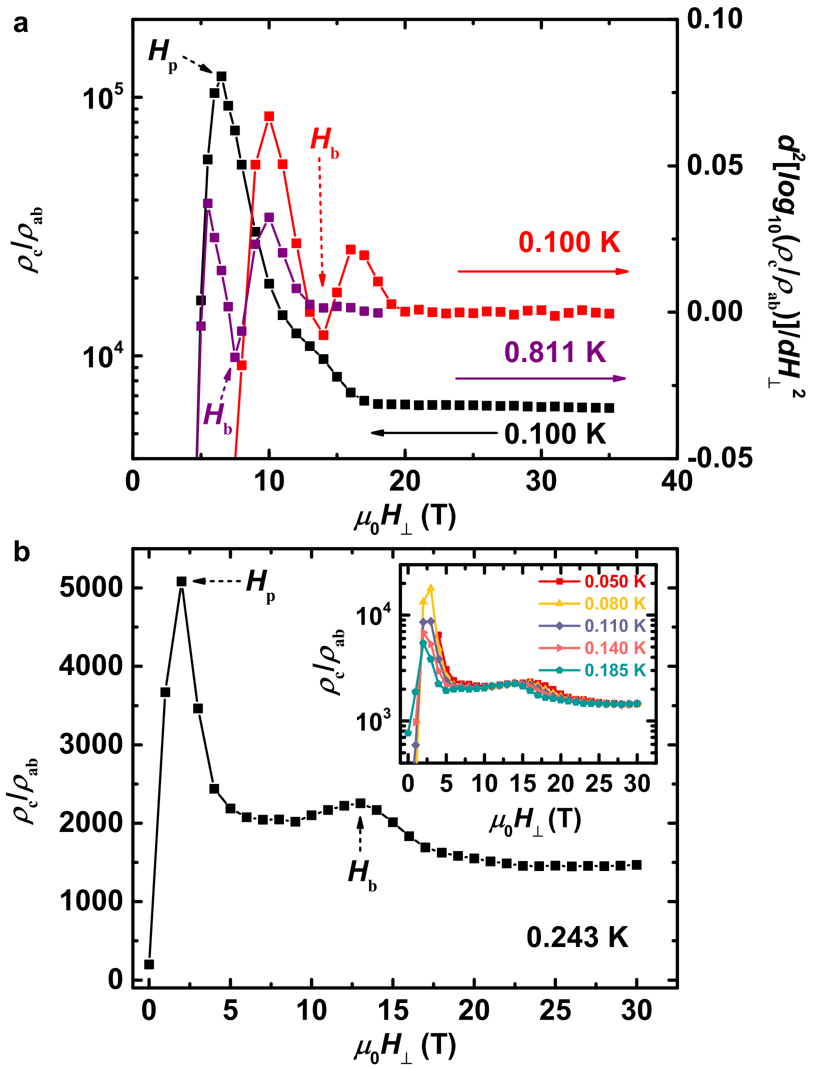

Supplementary Fig. 1: **Methods to determine characteristic fields in the $\boldsymbol{H_\perp}$ dependence of the anisotropy ratio $\boldsymbol{\rho_c/\rho_{ab}}$. a** $La_{1.7}Eu_{0.2}Sr_{0.1}CuO_4$. The anisotropy ratio ρ_c/ρ_{ab} (black symbols, left axis) vs $H_\perp$ at $T = 0.100$ K on a semi-log scale. Red and purple symbols (right axis) show the second derivative $d^2[\log(\rho_c/\rho_{ab})]/dH_\perp^2$ for $T = 0.100$ K and $T = 0.811$ K, respectively. Solid lines guide the eye. H_b is defined as the minimum in the second derivative, as shown. Clearly, H_b remains strongly pronounced even at a fairly high T. The analysis was repeated for different T. **b** $La_{1.48}Nd_{0.4}Sr_{0.12}CuO_4$; ρ_c/ρ_{ab} at $T = 0.243$ K. Although the absolute value of the anisotropy is relatively low, as noted previously[1] for La-214 cuprates with $x \approx 1/8$, the enhancement of ρ_c/ρ_{ab} at H_b is clearly observed already in the raw data. Inset: ρ_c/ρ_{ab} vs $H_\perp$ for several T.

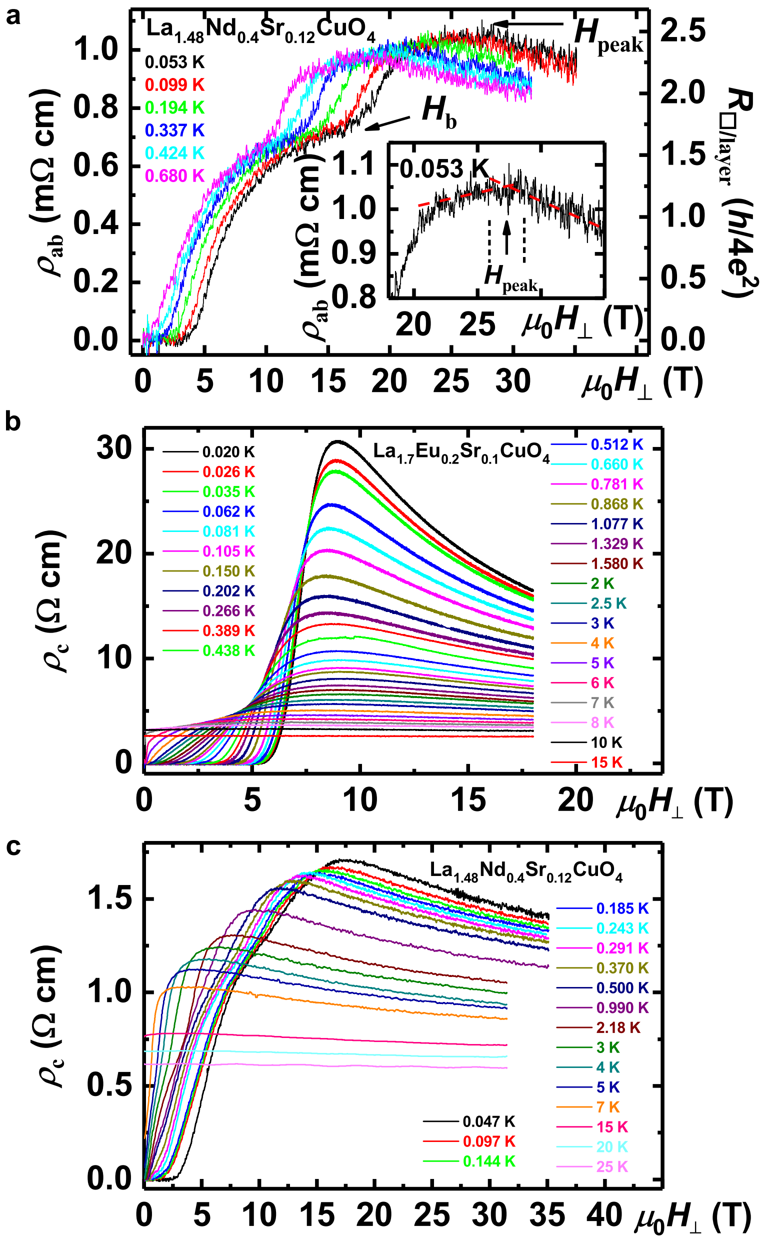

Supplementary Fig. 2: **The dependence of the in-plane and out-of-plane resistivity on $\boldsymbol{H_\perp}$.** **a** ρ_{ab} vs $H_\perp$ at several $T < T_c^0$ in $La_{1.48}Nd_{0.4}Sr_{0.12}CuO_4$. At low T, $\rho_{\mathrm{ab}}(H_\perp)$ exhibits a peak at $H_\perp = H_{\mathrm{peak}}(T)$. $H_{\mathrm{b}}(T)$ corresponds to the establishment of SC correlations in the planes, as the SC transition is approached from a high-field normal state; see also Figs. 1 and 2. The right axis shows the corresponding $R_{\square/\mathrm{layer}}$ in units of quantum resistance for Cooper pairs, $R_{\mathrm{Q}} = h/(2e)^2$. Inset: Determination of H_{peak} at $T = 0.053$ K. The vertical dashed lines indicate the uncertainty in estimating H_{peak} within the experimental resolution. **b** and **c** ρ_{c} vs $H_\perp$ for several T in $La_{1.7}Eu_{0.2}Sr_{0.1}CuO_4$ and $La_{1.48}Nd_{0.4}Sr_{0.12}CuO_4$, respectively.

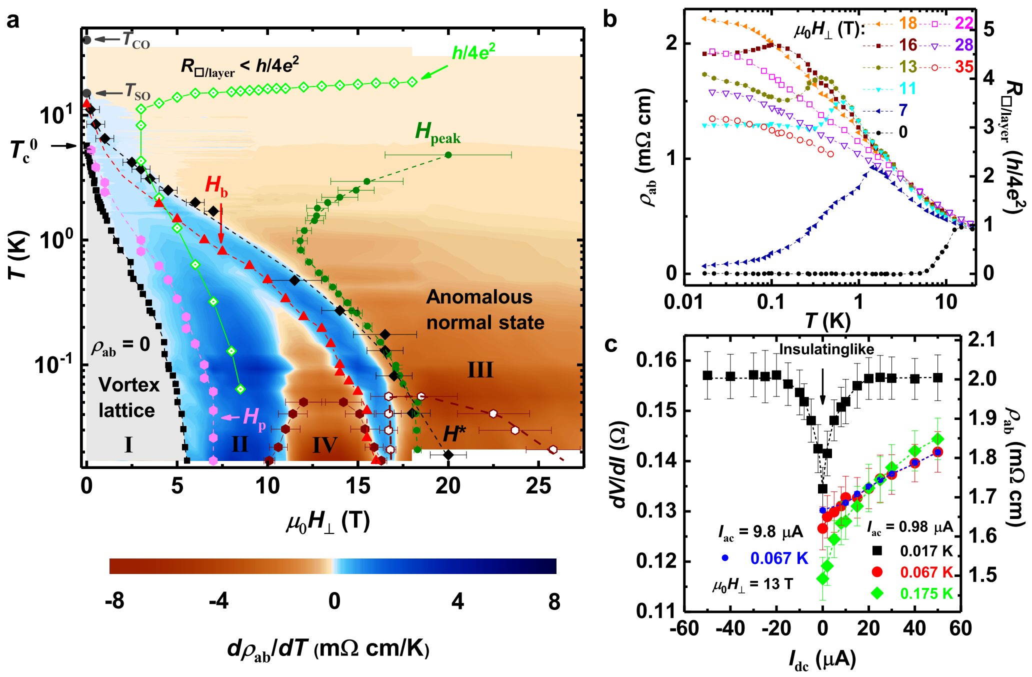

Supplementary Fig. 3: ***T–$H_\perp$* phase diagram of $La_{1.7}Eu_{0.2}Sr_{0.1}CuO_4$. a** Black squares: $T_c(H_\perp)$; $\rho_{ab} = 0$ for all $T < T_c(H_\perp)$ (region I). Color map: $d\rho_{ab}/dT$; in the viscous vortex liquid (II), $T_c = 0$. Dark brown dots: regime in which the MR hysteresis, independent of the field sweep rate, is observed. Dark brown open dots: the boundary of the hysteretic regime observed with a 1 T min^{-1} sweep rate (Supplementary Fig. 6a); the error bars reflect the uncertainty in ρ_{ab} due to T fluctuations and the experimental resolution for estimating the onsets of bifurcation. Green dots: $H_{peak}(T) \sim H_{c2}(T)$; as in Fig. 2, the error bars reflect the uncertainty in defining the MR peak within our experimental resolution (see Supplementary Fig. 2a inset for an example; also see Supplementary Fig. 6a and ref. 4 for the raw MR data). Black diamonds: $H^*(T)$, the boundary between non-ohmic V–I for $H_\perp < H^*$ and ohmic behavior found at $H_\perp > H^*$; error bars reflect the uncertainty of determining H^* within experimental resolution (also see ref. 4). Region III: $H_\perp$-revealed normal state. Open green diamonds: the $h/4e^2$ line. Pink dots: $H_p(T)$; red triangles: $H_b(T)$. $T_{SO}(H = 0)$ and $T_{CO}(H = 0)$ are also shown; both spin and charge stripes are known to be enhanced by $H_\perp$. **b** $\rho_{ab}(T)$ for several $0 \leq H_\perp \leq 35$ T. $d\rho_{ab}/dT < 0$ in region IV, e.g. for $H_\perp = 13$ T and $T < 0.1$ K, is comparable to that found in the normal state ($H_\perp > 20$ T), e.g. for $H_\perp = 28$ T and $H_\perp = 35$ T. In region III, $\rho_{ab} \propto \ln(1/T)$ is obeyed[23] at least down to ~ 0.06–0.07 K ("Methods"). **c** dV/dI vs I_{dc} for several T at $H_\perp = 13$ T (region IV); $I_{ac} \approx 1$ μA, but the data taken at $T = 0.067$ K show that the same result is obtained, within the error, with $I_{ac} \approx 1$ μA and $I_{ac} \approx 10$ μA. For each value of I_{dc}, the error bar is 1 SD obtained from averaging the ac voltage over 300 s ("Methods"; also ref. 4). The T-dependence of the linear resistance (dV/dI for $I_{dc} \to 0$) is insulatinglike. In all panels, dashed lines guide the eye.

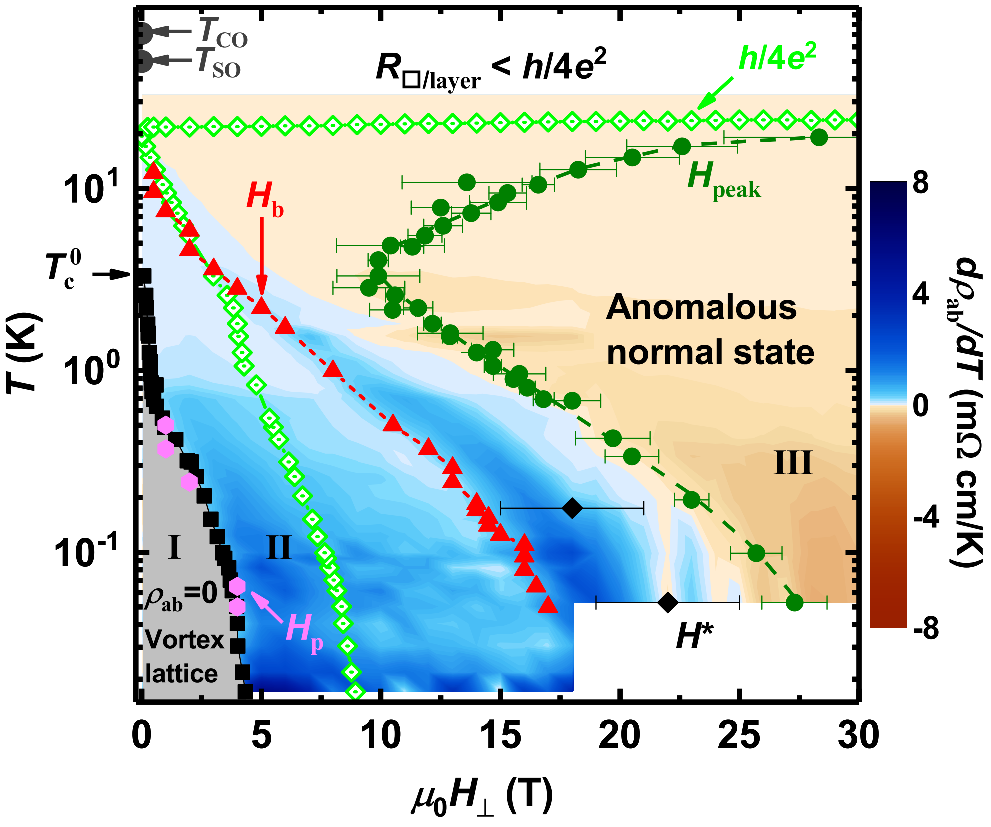

Supplementary Fig. 4: In-plane transport T–$H_\perp$ phase diagram of $La_{1.48}Nd_{0.4}Sr_{0.12}CuO_4$. Black squares: $T_c(H)$; $\rho_{ab} = 0$ for all $T < T_c(H)$ [region I; $T_c(H) > 0$]. The color map: slopes $d\rho_{ab}/dT$, clearly indicating a weakening (lighter blue color) of the metalliclike behavior at intermediate fields, i.e. within the viscous vortex liquid, for which $T_c = 0$ (region II). $H_{\text{peak}}(T) \sim H_{c2}(T)$ (green dots) represent fields above which the MR changes from positive to negative. Region III: $H_\perp$-revealed normal state. Open green diamonds: the $h/4e^2$ line. Pink dots: $H_p(T)$; red triangles: $H_b(T)$. In contrast to $La_{1.7}Eu_{0.2}Sr_{0.1}CuO_4$, here the layer decoupling field $H_p(T) \gtrsim H_c(T)$ (black squares), consistent with a stronger stripe order for $x \approx 1/8$. As in $La_{1.7}Eu_{0.2}Sr_{0.1}CuO_4$, the boundary of the weakened $\rho_{ab}(T)$ at intermediate fields is outlined by H_b and, roughly, by the $h/4e^2$ line. These results suggest that the insulatinglike regime would emerge at even lower, experimentally inaccessible T, in the $\sim 10-20$ T field range. Black diamonds: $H^*(T)$ represent the boundary[4] between non-ohmic V–I for $H_\perp < H^*$ and ohmic behavior found at $H_\perp > H^*$. The error bars for H_{peak} and H^* are defined in the same way as those for LESCO (see Fig. 2, Supplementary Figs. 2 and 3; also ref. 4). All dashed lines guide the eye. $T_{SO}(H = 0)$ and $T_{CO}(H = 0)$ are also shown; both spin and charge stripes are known to be enhanced by $H_\perp$ (see main text).

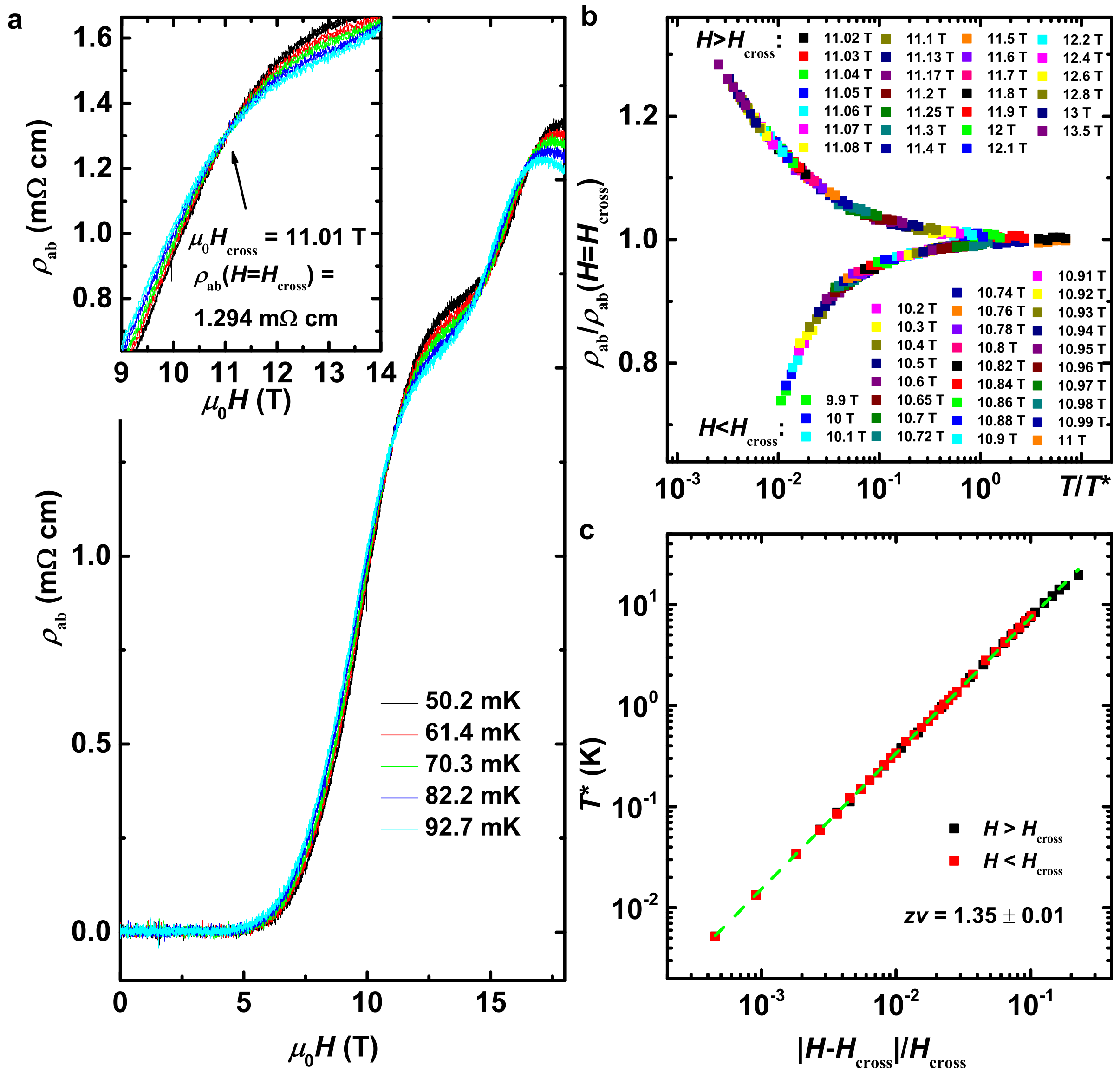

Supplementary Fig. 5: **Scaling of $\rho_{ab}(T, H)$ near the onset of region IV in Supplementary Fig. 3a in $La_{1.7}Eu_{0.2}Sr_{0.1}CuO_4$; $H \equiv H_\perp$.** **a** Isothermal $\rho_{ab}(H)$ curves at low T show the existence of a T-independent crossing point (inset) at $\mu_0 H_{cross} = 11.01$ T and $\rho_{ab}(H = H_{cross}) = 1.294$ mΩcm (or $R_{\square/\mathrm{layer}} \approx 3\ h/4e^2$). **b** Scaling of the data in **a** with respect to a single variable T/T^*; here, $\rho_{ab}(T, H) = \rho_{ab}(H = H_{cross}) f(T/T^*)$, i.e. the resistivity data for different H can be collapsed onto a single function by rescaling the temperature. **c** The scaling parameter T^* as a function of $|\delta| = |H - H_{cross}|/H_{cross}$ on both sides of H_{cross}. The dashed line is a linear fit with the slope $z\nu = 1.35 \pm 0.01$, as shown; $T^* \propto |\delta|^{z\nu}$.

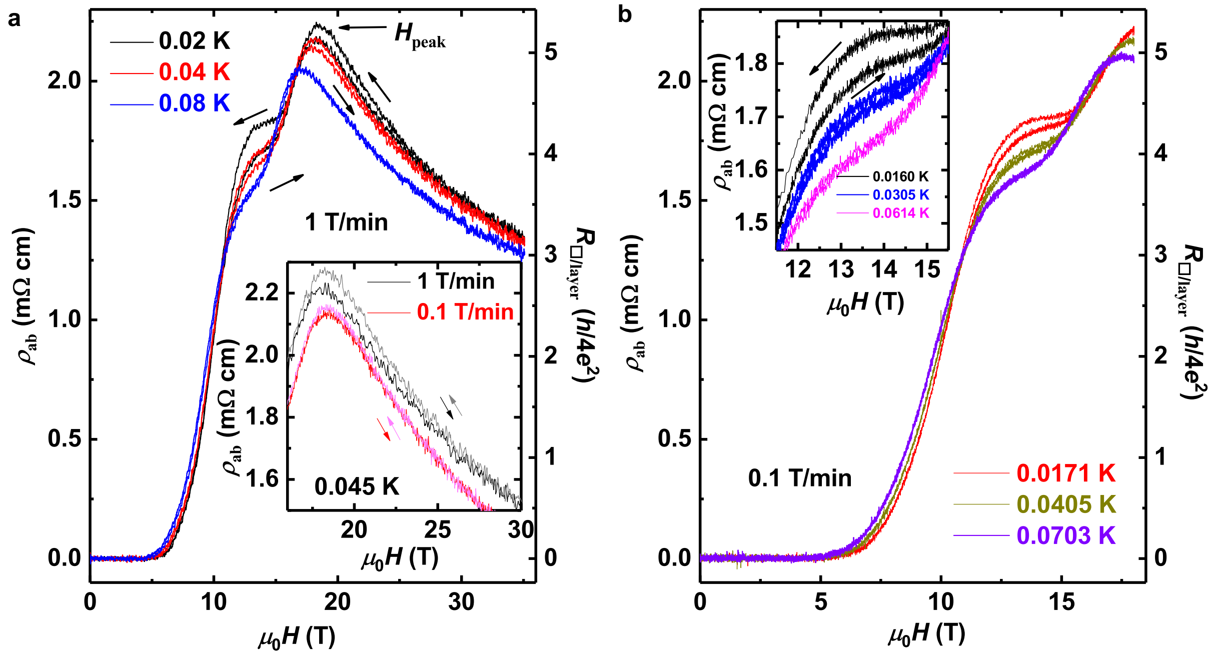

Supplementary Fig. 6: **In-plane resistivity of $La_{1.7}Eu_{0.2}Sr_{0.1}CuO_4$ vs H ∥ c. a** At low T, $\rho_{ab}(H)$ exhibits a sharp peak at $H = H_{peak}(T)$ and two hysteretic regimes: one occurs near a shoulder below the peak (region IV in Supplementary Fig. 3a) and the other starts at $H \sim H_{peak}$. The width in H of the lower-field hysteretic region is the same for sweep rates between 1 T min^{-1}, shown here, and 0.1 T min^{-1} (see **b**). Inset: The higher-field hysteresis is less robust, as its width is reduced with decreasing sweep rate. The 0.1 T min^{-1} trace, which shows a small hysteresis near H_{peak}, is shifted down by 0.12 mΩ cm for clarity. Arrows show the direction of field sweeps. **b** The hysteretic, insulatinglike region IV is surrounded by the regimes of metallic behavior. Inset: The hysteresis is suppressed with increasing T.

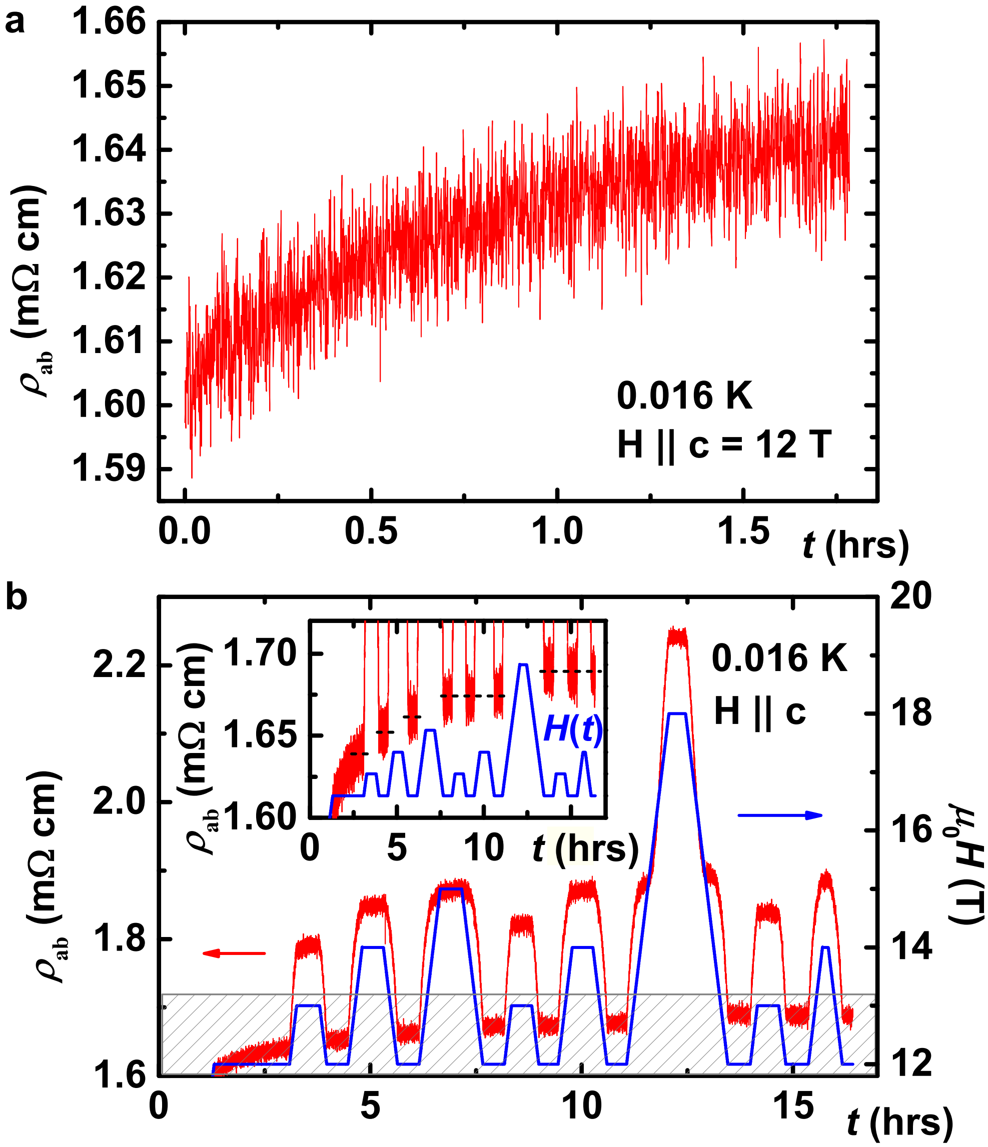

Supplementary Fig. 7: **Nonequilibrium dynamics in region IV of the $La_{1.7}Eu_{0.2}Sr_{0.1}CuO_4$ phase diagram in Supplementary Fig. 3a.** **a** ρ_{ab} exhibits slow, nonexponential relaxations with time t: here it continues to relax for hours after the magnetic field reaches 12 T at $T = 0.016$ K. **b** At a fixed $T = 0.016$ K, ρ_{ab} (red; left axis) is measured as a function of time as $H_\perp$ is changed between 12 T and different higher fields (blue; right axis). This protocol allows a comparison of ρ_{ab} values obtained at the same $\mu_0 H_\perp = 12$ T but with a different magnetic history. Inset: Enlarged shaded area of the main plot shows that $\rho_{ab}(\mu_0 H_\perp = 12\text{ T})$ is determined by the highest $H_\perp$ applied previously: the system acquires a memory of its magnetic history. Dashed lines guide the eye.

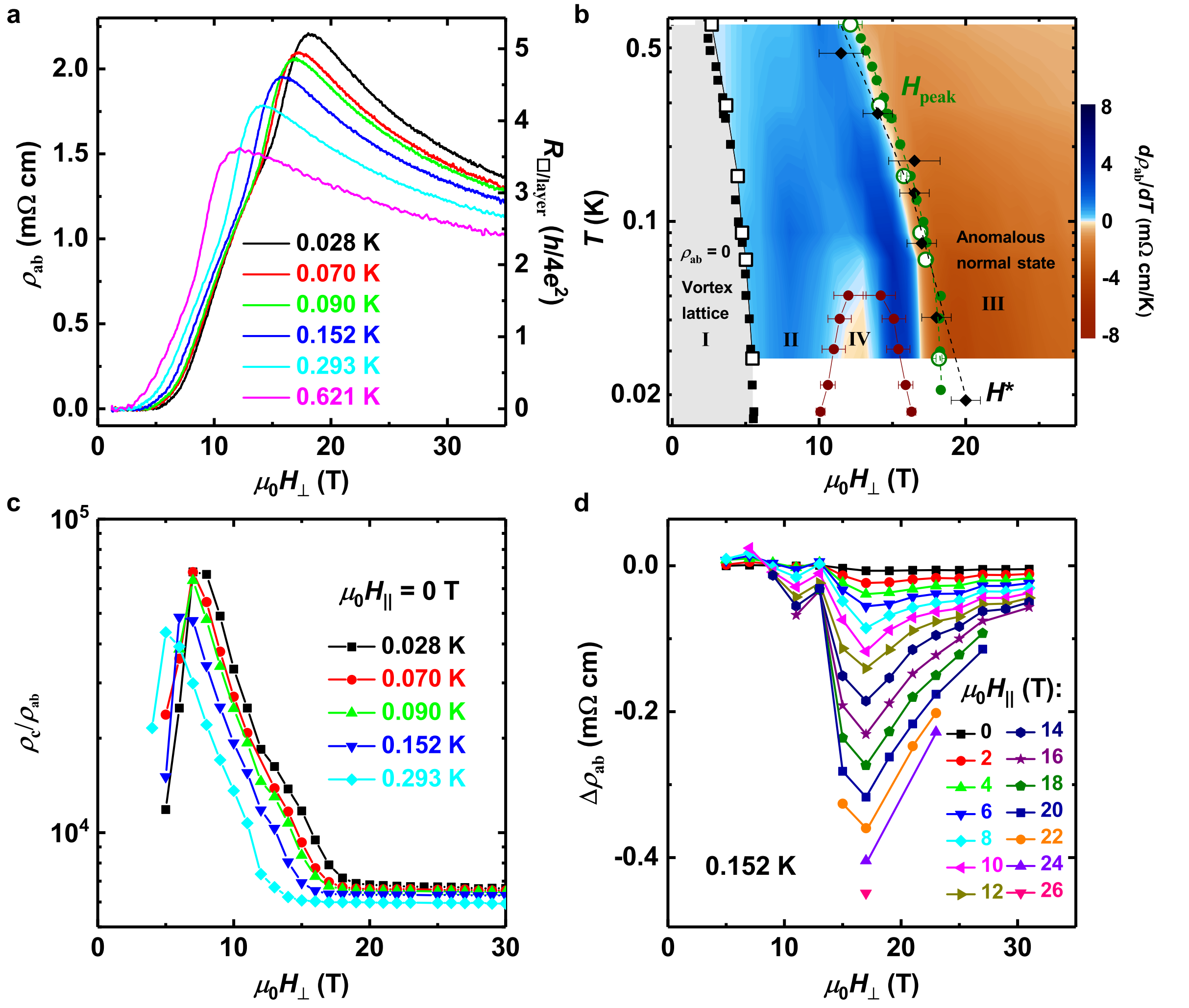

Supplementary Fig. 8: **In-plane $La_{1.7}Eu_{0.2}Sr_{0.1}CuO_4$ sample B1.** **a** ρ_{ab} vs $H_\perp$ (i.e. $\mathbf{H} \parallel \mathbf{c}$) for several T, as shown. **b** In-plane transport T–H phase diagram with $\mathbf{H} \parallel \mathbf{c}$ axis. The color map shows $d\rho_{ab}/dT$ on the same scale as that in Supplementary Fig. 3a for sample B. Open black squares and open green dots represent $T_c(H)$ and $H_{peak}(T)$, respectively. For comparison, solid symbols show the corresponding values for sample B; dark brown dots show the boundary of the hysteretic regime (region IV) in sample B. While the values of $T_c(H)$ and $H_{peak}(T)$ in B and B1 match within error, the insulatinglike region IV is clearly suppressed to lower T in sample B1 but, at the same time, the reentrant vortex liquid regime is more pronounced. For completeness, solid diamonds show the values of $H^*(T)$, the boundary between non-ohmic and ohmic transport, for sample B. **c** ρ_c/ρ_{ab} vs H $\parallel$ c at different T, as shown. Solid lines guide the eye. **d** The suppression of the in-plane resistivity by $H_\parallel$, $\Delta\rho_{ab} = \rho_{ab}(H_\parallel) - \rho_{ab}(H_\parallel = 0)$, for different $H_\parallel$, as shown, as a function of $H_\perp$ at $T = 0.152$ K. Solid lines guide the eye.

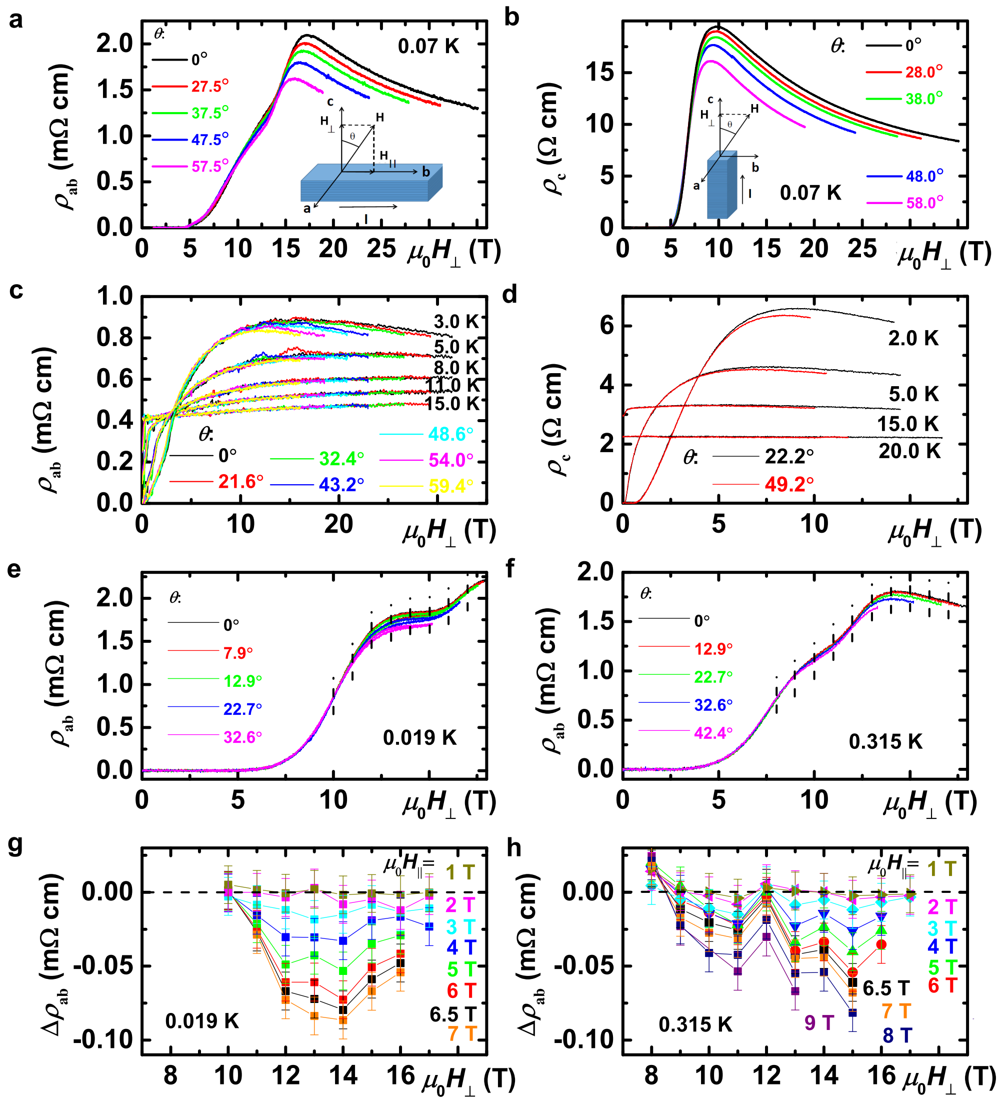

Supplementary Fig. 9: **Angle-dependent transport in $La_{1.7}Eu_{0.2}Sr_{0.1}CuO_4$ vs $\boldsymbol{H_\perp}$.** **a** ρ_{ab} (sample B1), and **b** ρ_c, at $T = 0.07$ K and for different angles θ, as shown; the uncertainty $\Delta\theta = 0.5°$. The sketches show the field orientation with respect to the sample axes and the current flow. **c** ρ_{ab} (sample B), and **d** ρ_c, at higher T and for different angles θ, as shown. While the effect of $H_\parallel$ on ρ_{ab} vanishes at ~ 5 K, i.e. at $T \sim T_c^0$ (**c**), in ρ_c it vanishes at ~ 15 K, i.e. at $T \sim T_{SO}$ (**d**); in both cases, this seems to be related to the vanishing of the peak in the MR. **e-h** Sample B. $\rho_{ab}(H_\perp)$ for different angles θ, as shown, at $T = 0.019$ K (**e**) and $T = 0.315$ K (**f**). Vertical dashed lines indicate the values of $H_\perp$ used in figures **g** and **h**. **g** and **h** show $\Delta\rho_{ab} = \rho_{ab}(H_\parallel) - \rho_{ab}(H_\parallel = 0)$, i.e. the effect of the in-plane fields $H_\parallel$, as shown, on $\rho_{ab}(H_\perp)$ at $T = 0.019$ K and $T = 0.315$ K, respectively. The error bars reflect the uncertainty caused by the finite experimental resolution of $\rho_{ab}(H)$. Thin solid lines guide the eye.

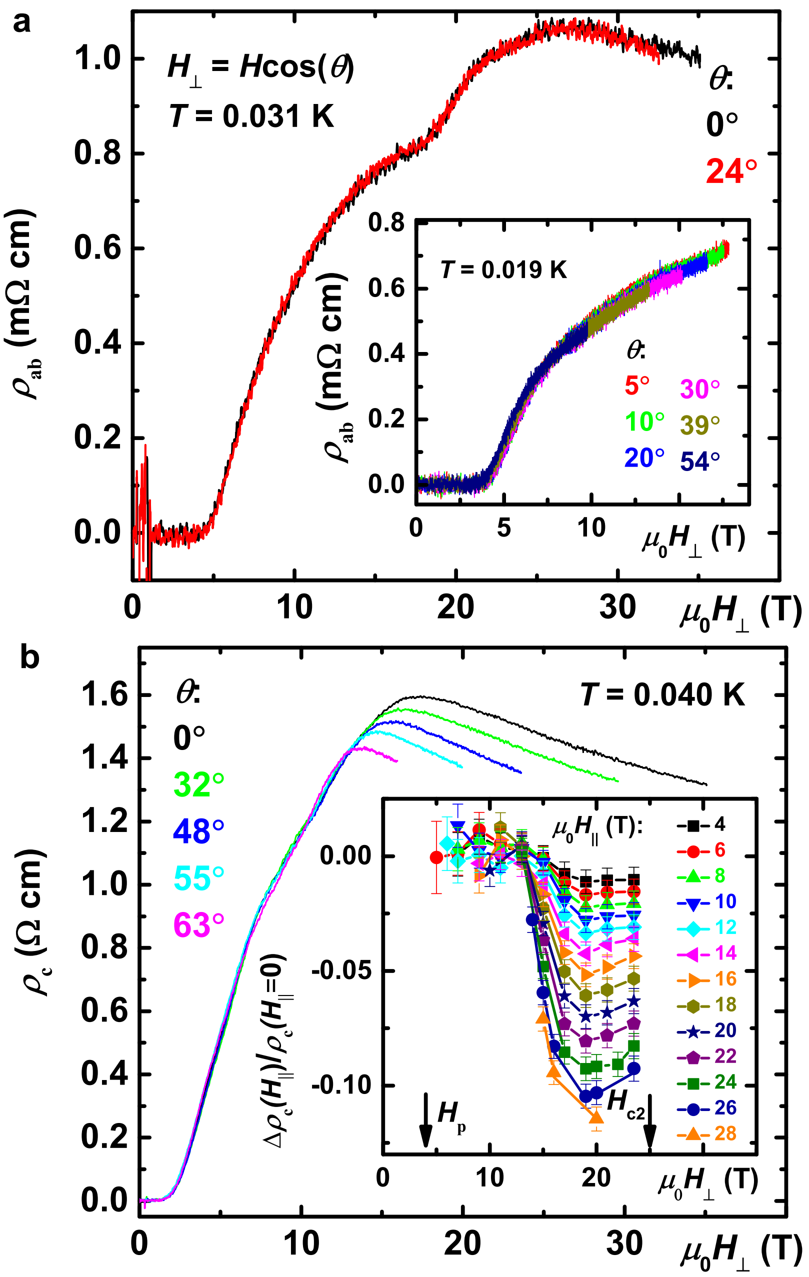

Supplementary Fig. 10: **Angle-dependent transport in $La_{1.48}Nd_{0.4}Sr_{0.12}CuO_4$ vs $\boldsymbol{H_\perp}$ at low $\boldsymbol{T}$. a** ρ_{ab}, and **b** ρ_c, for different angles θ and T, as shown; the uncertainty $\Delta\theta = 0.5°$. The in-plane $H_\parallel = H\sin\theta$ does not affect ρ_{ab}; here $H_\parallel$ was oriented parallel to the crystallographic [110] (or $[1\bar{1}0]$) axis. On the other hand, ρ_c is reduced by $H_\parallel$; here the field was parallel to the crystallographic a (or b) axis. The inset in **b** shows the corresponding $\Delta\rho_c(H_\parallel)/\rho_c(H_\parallel = 0) = \rho_c(H_\parallel)/\rho_c(H_\parallel = 0) - 1$; $T = 0.040$ K. The error bars reflect the uncertainty caused by the finite experimental resolution of $\rho_c(H)$.

Supplementary Note 1

Superconducting transition temperature and vortex phase diagram

$T_c(H_\perp)$ were determined as the temperatures at which the linear resistance $R \equiv \lim_{I_{dc}\to 0} V/I$ (or resistivity) becomes zero (V – voltage, I – current). In both $La_{1.7}Eu_{0.2}Sr_{0.1}CuO_4$ and $La_{1.48}Nd_{0.4}Sr_{0.12}CuO_4$, ρ_c and ρ_{ab} vanish at the same temperature within the error, indicating the onset of 3D superconductivity. In contrast, in striped $La_{1.875}Ba_{0.125}CuO_4$ in $H = 0$, a 2D superconductivity was reported[2] to appear at a T higher than the onset of 3D superconductivity, although it has been suggested[3] that higher precision measurements might reveal the same T_c^0 for both ρ_c and ρ_{ab}.

The position of the peak in $\rho_{ab}(H_\perp)$ (see, e.g., Supplementary Fig. 2a), $H_\perp = H_{peak}(T)$, was found[4] to be of the order of the upper critical field (H_{c2}), i.e. the field scale corresponding to the closing of the SC gap. Therefore, the superconductor with $T_c(H) > 0$ (i.e. a $\rho_{ab} = 0$ state) is separated from the normal state at $H_\perp > H_{peak}$ by a wide regime of SC phase fluctuations arising from the motion of vortices. At low T, this regime exhibits non-ohmic transport[4] consistent with the motion of vortices in the presence of disorder: it was thus identified[4] as a viscous vortex liquid with the zero freezing temperature, i.e. $T_c = 0$.

Supplementary Note 2

In-plane transport in perpendicular magnetic fields

The in-plane T–$H_\perp$ phase diagrams of $La_{1.7}Eu_{0.2}Sr_{0.1}CuO_4$ and $La_{1.48}Nd_{0.4}Sr_{0.12}CuO_4$ are shown in Supplementary Figs. 3a and 4, respectively. In order to display $\rho_{ab}(T)$ for all $H_\perp$, we use the color maps. The metalliclike, $d\rho_{ab}/dT > 0$ regions where $\rho_{ab}(T > 0) \neq 0$ (blue regions II in Supplementary Figs. 3a and 4), which exhibit non-ohmic transport at low T, were identified[4] as a viscous vortex liquid with the zero freezing temperature, i.e.

$T_c = 0$.

In $La_{1.7}Eu_{0.2}Sr_{0.1}CuO_4$, within the phase-fluctuations regime, there is clearly a region of pronounced insulatinglike ($d\rho_{ab}/dT < 0$) behavior at low T (region IV in Supplementary Fig. 3a), with its precursors, i.e. the weakening of the metalliclike T dependence, becoming visible already at $T \lesssim T_c^0$ (see also Supplementary Fig. 4 for $La_{1.48}Nd_{0.4}Sr_{0.12}CuO_4$), i.e. at $H_b(T)$ (see also Fig. 2). The insulatinglike $d\rho_{ab}/dT$ that develops at low T, in region IV, is at least as strong as the one observed in the field-revealed normal state, i.e. for $H_\perp > H_{c2} \sim 20$ T (Supplementary Fig. 3b). By tracking the "$h/4e^2$" line where $R_{\square/\mathrm{layer}}$ changes from $R_{\square/\mathrm{layer}} < R_Q$ at lower $H_\perp$ and higher T, to $R_{\square/\mathrm{layer}} > R_Q$ at higher $H_\perp$ and lower T, we find that it has two branches (Supplementary Figs. 3a and 4): while the upper one seems to form an upper limit for the presence of vortices[4], the lower one extrapolates roughly to the onset of region IV as $T \to 0$, suggesting that region IV may be related to the localization of Cooper pairs. Indeed, although the range of T and $H_\perp$ is limited, the scaling behavior of $\rho_{ab}(T, H_\perp)$ near the onset of region IV (Supplementary Fig. 5) seems consistent with the presence of a $T = 0$ SIT driven by quantum phase fluctuations in a disordered 2D system[5].

However, the V–I measurements in region IV reveal a non-ohmic increase of dV/dI with I_{dc} (Supplementary Fig. 3c), in contrast to the observations[6] on the insulating side of the 2D SIT where dV/dI decreases with I_{dc}. On the other hand, a non-ohmic increase of dV/dI with I_{dc} is consistent with the motion of vortices in the presence of disorder (i.e. a viscous vortex liquid)[4,7]. The increase of dV/dI with I_{dc} is precisely the opposite of what would be expected in the case of simple Joule heating, confirming the presence of SC correlations, characteristic of a vortex liquid, in region IV. Our results thus strongly suggest that region IV consists of SC puddles, with no inter-puddle phase coupling, in an insulatinglike, high-field normal-state background: at low T, the increasing $H_\perp$ destroys

the superconductivity in the planes by quantum phase fluctuations of Josephson-coupled SC puddles. The evolution of this region with T can be traced to the initial, metalliclike drop of $\rho_{\rm ab}(T)$ at $T > T_{\rm c}^0$ in $H = 0$ (see also $H_{\rm b}$ dashed line in Figs. 2c and 2d). In $La_{1.48}Nd_{0.4}Sr_{0.12}CuO_4$, the lower branch of the "$h/4e^2$" line, together with $H_{\rm b}(T)$, practically outlines the region of the weakened, metalliclike $\rho_{\rm ab}(T)$. Although the insulatinglike behavior is not observed, these results strongly suggest that it would ultimately emerge at even lower, experimentally inaccessible T, roughly in the $\sim 10-20$ T field range, similar to $La_{1.7}Eu_{0.2}Sr_{0.1}CuO_4$.

The weakening of the metalliclike T dependence at intermediate $H_{\perp}$, which leads to the insulatinglike behavior in $La_{1.7}Eu_{0.2}Sr_{0.1}CuO_4$ at low T (region IV in Supplementary Fig. 3), is manifested by the appearance of a "shoulder" in the in-plane MR curves for $H < H_{\rm peak}$ (Supplementary Figs. 6a and 2a). The shoulder in the MR becomes more noticeable with decreasing T and, at very low $T \lesssim 0.05$ K, the MR in this range of fields becomes hysteretic (Supplementary Fig. 6a). The size of the hysteresis grows with decreasing T (Supplementary Fig. 6b), and the range of fields where it is observed, independent of the sweep rate, outlines the boundary of region IV (Supplementary Fig. 3a) where $d\rho_{\rm ab}/dT < 0$. In other words, the hysteretic MR is not observed at even higher $H_{\perp}$, where $d\rho_{\rm ab}/dT > 0$ (blue sliver in Supplementary Fig. 3a). Another hysteretic regime appears as the system enters the normal state (Supplementary Fig. 6a), but it is much less robust: its width in $H_{\perp}$ is reduced with decreasing sweep rate (Supplementary Fig. 6a inset; Supplementary Fig. 3a shows the boundaries corresponding to 1 T/min). In general, a hysteresis is a manifestation of the coexistence of phases, i.e. it indicates the presence of domains of different phases in the system. Typical signatures of such systems include slow, nonexponential relaxations and memory effects, which are indeed observed here (Supplementary Fig. 7). The hysteretic response to $H_{\perp}$, observed when the super-

conductivity is suppressed, is attributed to the presence of domains with spin stripes.

Supplementary Note 3

Effects of parallel magnetic fields on the spin structure

In contrast to $La_{2-x}Ba_xCuO_4$, the magnetization of $La_{1.8-x}Eu_{0.2}Sr_xCuO_4$ and $La_{1.6-x}Nd_{0.4}Sr_xCuO_4$ is dominated by the rare-earth ion (e.g. refs. 8, 9), so that studies of the Cu spin magnetism are difficult and scarce in these compounds. Nevertheless, it is known that, in the low-temperature tetragonal phase of antiferromagnetic $La_{1.8}Eu_{0.2}CuO_4$, an in-plane magnetic field ($\mathbf{H} \parallel$ b) of ~ 6 T leads to a spin-flop transition[8] of the Cu spin moments in every other plane. Moreover, this spin-flop transition field is roughly the same as that in $La_{1.875}Ba_{0.125}CuO_4$ for $T < T_{\mathrm{SO}}$ (ref. 10). Since the structure of these three materials is similar[11], it is thus likely that the spin-flop transition occurs also in $La_{1.8-x}Eu_{0.2}Sr_xCuO_4$ and $La_{1.6-x}Nd_{0.4}Sr_xCuO_4$ near $x = 1/8$ at comparable fields. $La_{2-x}Sr_xCuO_4$ with $x = 0.115$, for example, also exhibits a spin-flop transition at a similar field[12] ≈ 7.5 T. We note that, in case of $\mathbf{H} \parallel [110]$, the transition is broader: spins in all planes continuously rotate until staggered moment is again perpendicular to the field, so there is no sharp spin-flop transition[10,13]. In all cases, however, the reorientation of spins under the influence of an in-plane field ($\mathbf{H} \parallel$ ab) should enhance Josephson coupling between layers, within the PDW picture.

In the $\mathbf{H} \parallel [110]$ configuration, Josephson coupling between layers may be enhanced also by another mechanism, namely by the field partially compensating for the momentum mismatch between the layers[14]; that mechanism would reduce ρ_{c}, but it would have no effect on ρ_{ab}. In our in-plane $La_{1.48}Nd_{0.4}Sr_{0.12}CuO_4$ crystal, which was cut at a 45° angle with respect to a and b axes ("Methods"), we do not see, indeed, any observable

effect of $\mathbf{H} \parallel [110]$ on ρ_{ab} (Supplementary Fig. 10a). This also implies that, in contrast to $La_{1.7}Eu_{0.2}Sr_{0.1}CuO_4$, (Fig. 3c, top), the effect of spin reorientation on ρ_{ab} is too weak to be observed within the experimental resolution. We note that, at the same time, in our out-of-plane $La_{1.48}Nd_{0.4}Sr_{0.12}CuO_4$ crystal for which $\mathbf{H} \parallel [100]$ ("Methods") and in which the mechanism of ref. 14 thus cannot play a role, the reduction in ρ_{c} (Supplementary Fig. 10b) is also weaker than in $La_{1.7}Eu_{0.2}Sr_{0.1}CuO_4$. The weaker spin reorientation effect of $H_{\parallel}$ on ρ_{c} and ρ_{ab} in $La_{1.48}Nd_{0.4}Sr_{0.12}CuO_4$ than in $La_{1.7}Eu_{0.2}Sr_{0.1}CuO_4$ is, therefore, attributed to the stronger pinning of stripe order at $x = 1/8$.

Supplementary References

1. Berg, E., Fradkin, E., Kivelson, S. A. & Tranquada, J. M. Striped superconductors: how spin, charge and superconducting orders intertwine in the cuprates. New J. Phys. **11,** 115004 (2009).

2. Li, Q., Hücker, M., Gu, G. D., Tsvelik, A. M. & Tranquada, J. M. Two-dimensional superconducting fluctuations in stripe-ordered $La_{1.875}Ba_{0.125}CuO_4$. *Phys. Rev. Lett.* **99,** 067001 (2007).

3. Berg, E., Fradkin, E. & Kivelson, S. A. Theory of the striped superconductor. *Phys. Rev. B* **79,** 064515 (2009).

4. Shi, Z., Baity, P. G., Sasagawa, T. & Popović, D. Vortex phase diagram and the normal state of cuprates with charge and spin orders. *Sci. Adv.* **6,** eaay8946 (2020).

5. Fisher, M. P. A. Quantum phase transitions in disordered two-dimensional superconductors. *Phys. Rev. Lett.* **65,** 923 (1990).

6. Qin, Y., Vicente, C. L. & Yoon, J. Magnetically induced metallic phase in superconducting tantalum films. *Phys. Rev. B* **73,** 100505(R) (2006).

7. Giamarchi, T. Disordered Elastic Media. *Encyclopedia of Complexity and Systems Science.* (Ed. R. A. Meyers, Springer, New York, 2009).

8. Hücker, M. *et al.* Dzyaloshinsky-Moriya spin canting in the low-temperature tetragonal phase of $La_{2-x-y}Eu_ySr_xCuO_4$. *Phys. Rev. B* **70,** 214515 (2004).

9. Hücker, M. Electronic interlayer coupling in the low-temperature tetragonal phase of $La_{1.79}Eu_{0.2}Sr_{0.001}CuO_4$. *Phys. Rev. B* **79,** 104523 (2009).

10. Hücker, M., Gu, G. D. & Tranquada, J. M. Spin susceptibility of underdoped cuprate superconductors: Insights from a stripe-ordered crystal. *Phys. Rev. B* **78,** 214507 (2008).

11. Hücker, M. Structural aspects of materials with static stripe order. *Physica C* **481,** 3–14 (2012).

12. Chiba, K., Goto, T., Mori, M., Suzuki, T., Seki, K. & Fukase, T. ^{139}La-NMR study of spin-flop and spin structure in $La_{2-x}Sr_xCuO_4$ $(x \sim 1/8)$. *J. Low Temp. Phys.* **117,** 479–483 (1999).

13. Baek, S.-H. *et al.* Magnetic field induced anisotropy of ^{139}La spin-lattice relaxation rates in stripe ordered $La_{1.875}Ba_{0.125}CuO_4$. *Phys. Rev. B* **92,** 155144 (2015).

14. Yang, K. Detection of striped superconductors using magnetic field modulated Josephson effect. *J. Supercond. Nov. Magn.* **26,** 2741–2742 (2013).
