# Supplementary material for: Pair density wave at high magnetic fields in cuprates with charge and spin orders
Source: Nat Commun. 2020 Jul 3;11:3323. doi: 10.1038/s41467-020-17138-z (PMC7335199; doi:10.1038/s41467-020-17138-z)
Supplement: Supplementary file 2 — Peer Review File [file 41467_2020_17138_MOESM2_ESM.pdf]

Editorial Note: This manuscript has been previously reviewed at another journal that is not operating a transparent peer review scheme. This document only contains reviewer comments and rebuttal letters for versions considered at Nature Communications.

REVIEWERS' COMMENTS:

Reviewer #1 (Remarks to the Author):

This is a nice improvement of the earlier version: the presentation is more focused on new data consistent with the PDW scenario, the presentation is also simpler and more accessible to the general reader. I recommend publication.

Reviewer #2 (Remarks to the Author):

The authors have transferred this paper from Nature Physics to Nature Communications. The views of my previous report for Nature Physics applies in this case as well, with one key exception: I find this rewritten version much clearer and easier to follow than the previous version. As in the previous manuscript, and as also pointed out by Reviewer 4, the key issue in the interpretation of this work is the possibility of inhomogeneity. In this latest version I feel the authors have furthered strengthen their argument against this possibility by linking the two-step transition more closely to 1/8'th doping. I therefore feel this manuscript is suitable for publication in Nature Communications.
